# Targeting histone methylation for cancer therapy: enzymes, inhibitors, biological activity and perspectives

**DOI:** 10.1186/s13045-016-0279-9

**Published:** 2016-06-17

**Authors:** Yongcheng Song, Fangrui Wu, Jingyu Wu

**Affiliations:** Department of Pharmacology, Baylor College of Medicine, 1 Baylor Plaza, Houston, TX 77030 USA; Dan L. Duncan Cancer Center, Baylor College of Medicine, 1 Baylor Plaza, Houston, TX 77030 USA

**Keywords:** Histone methylation, Enzyme inhibitor, Histone lysine methyltransferase, Protein arginine methyltransferase, Histone demethylase, Cancer therapeutics

## Abstract

Post-translational methylation of histone lysine or arginine residues plays important roles in gene regulation and other physiological processes. Aberrant histone methylation caused by a gene mutation, translocation, or overexpression can often lead to initiation of a disease such as cancer. Small molecule inhibitors of such histone modifying enzymes that correct the abnormal methylation could be used as novel therapeutics for these diseases, or as chemical probes for investigation of epigenetics. Discovery and development of histone methylation modulators are in an early stage and undergo a rapid expansion in the past few years. A number of highly potent and selective compounds have been reported, together with extensive preclinical studies of their biological activity. Several compounds have been in clinical trials for safety, pharmacokinetics, and efficacy, targeting several types of cancer. This review summarizes the biochemistry, structures, and biology of cancer-relevant histone methylation modifying enzymes, small molecule inhibitors and their preclinical and clinical antitumor activities. Perspectives for targeting histone methylation for cancer therapy are also discussed.

## Background

Nucleosome is the smallest structural unit of the human genetic material, which is composed of ~146 base pairs of double-stranded DNA wrapped around a histone octamer that contains two copies of histone H2A, H2B, H3, and H4 proteins. Basic lysine and arginine residues are enriched in histones. At physiological pH, these positively charged sidechains provide strong electrostatic and H-bond interactions with the negatively charged DNA for tight binding and packaging. Chromatin, a linear array of millions of nucleosomes, is organized into higher orders of structure and tightly condensed to form a chromosome. Functionally, chromatin is classified into highly packed, transcriptionally inactive heterochromatin and transcriptionally active euchromatin, whose structure is less condensed and DNA is therefore more accessible to a transcription machinery [1, 2]. Post-translational modifications of histones, such as acetylation and methylation, largely control DNA accessibility and regulate gene expression. For example, acetylation can neutralize the positive charged lysine sidechain and render a more open DNA structure to facilitate the binding of transcription factors as well as other proteins for gene expression. Abnormal histone modifications often occur in many diseases such as cancer. Histone modifying enzymes are therefore potential drug targets for these diseases [3, 4]. Small molecule inhibitors of histone deacetylases (HDAC) have been extensively developed and several compounds, such as vorinostat and romidepsin, have been approved to treat T-cell lymphomas [5–7]. Moreover, HDAC inhibitors have been in many clinical trials for other hematologic and solid cancers, with >500 studies in clinicaltrials.gov.

Physiological and pathological functions of histone methylation in a lysine or arginine residue have been well studied and documented. These post-translational modifications play crucial roles in gene regulation, cell differentiation, DNA recombination, and damage repair in normal cells as well as pathogenesis in diseases [4, 8]. A large family of ≥60 histone methyltransferases (HMT), including histone lysine methyltransferases (HKMT) and protein/histone arginine methyltransferases (PRMT), were identified in humans, among which the biochemical and biological functions of many methyltransferases have been characterized [9, 10]. In addition, histone methylation is dynamically controlled by histone/protein lysine demethylases (KDM), enzymes that remove the methyl group(s) from a methylated lysine sidechain [11–13]. The opposite functions between HMTs and KDMs facilitate to maintain balanced histone methylation levels. Aberrant histone methylations have been frequently found in cancer [4, 8], caused by a gene mutation, translocation, or dysregulated expression. Therefore, many HMTs and KDMs are potential drug targets and small molecule inhibitors of these proteins are useful chemical probes or potential therapeutics. As compared to that of HDAC inhibitors, development of histone methylation modulators has been in an early stage [4, 14]. There were very few potent inhibitors of HMTs and KDMs before 2010. Significantly more efforts from the academia and pharmaceutical industry have been observed during the past few years, leading to a rapidly increased number of small molecule modulators of histone methylation [15–17]. Several potent and selective compounds have recently been in clinical trials against acute myeloid leukemia (AML), non-Hodgkin lymphoma and lung cancer, showing the great potential for this class of compounds in cancer therapy.

This review summarizes the biochemistry, structures, and biology of histone methylation modifying enzymes, small molecule inhibitors and their preclinical and clinical antitumor activities. Due to the large number of these proteins, only those highly relevant to cancer are described, with a particular focus on histone H3 lysine 79 (H3K79) methyltransferase DOT1L, H3K4 targeting mixed lineage leukemia (MLL) and lysine-specific demethylase 1 (LSD1), and H3K27 methyltransferase EZH2. We also include mutations of isocitrate dehydrogenases (IDH), which have recently been found in 20–80 % of gliomas, AML and several types of sarcomas. The mutant IDH proteins indirectly inhibit a broad range of histone demethylases and cause genome-wide histone hypermethylation. In addition, perspectives of targeting histone methylation for cancer therapy are discussed.

## Biochemistry and structure

**Fig. 1:**
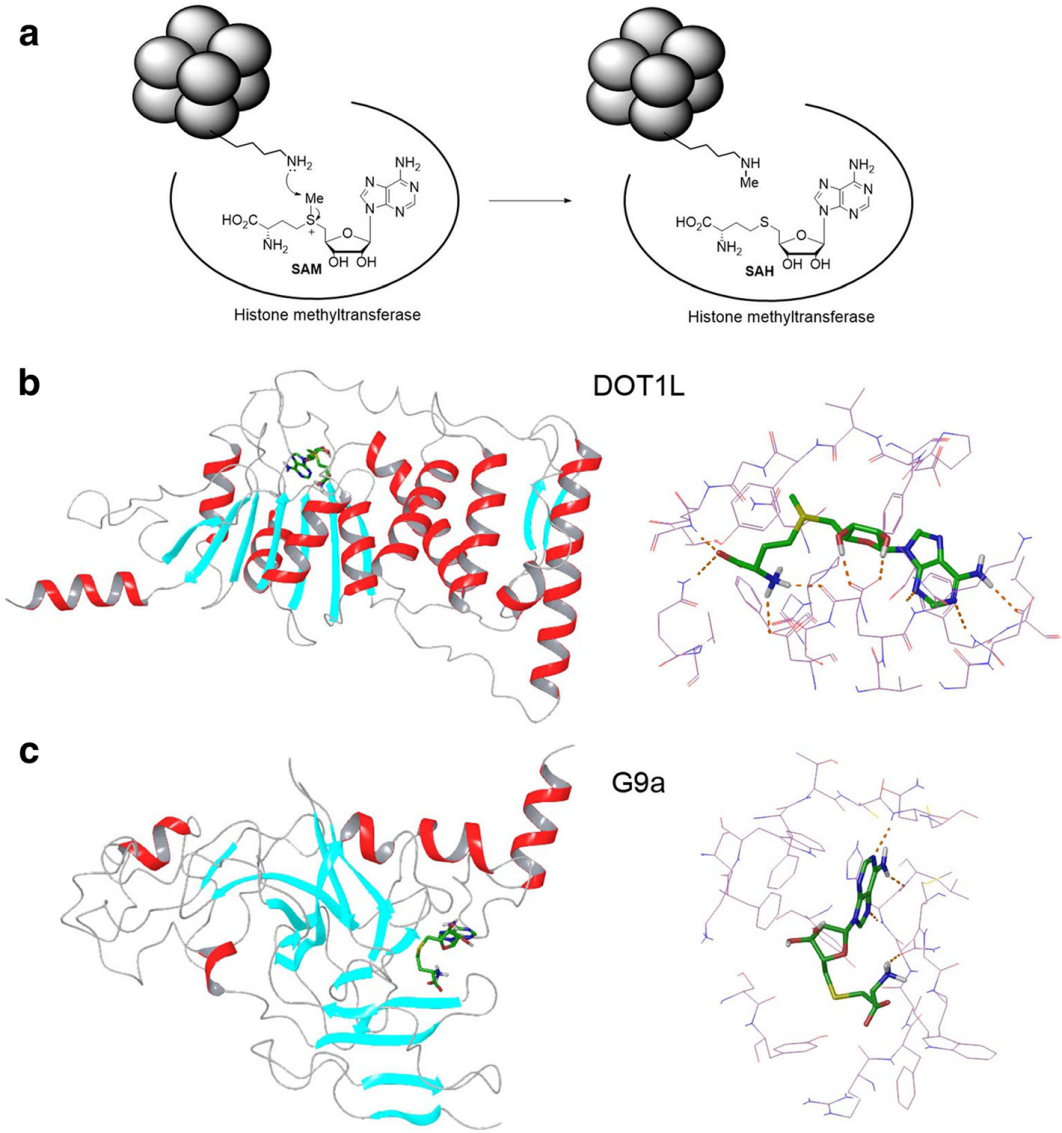
Mechanism and structures of histone methyltransferases (HMT). **a** Mechanism of catalysis for HMTs. Upon binding to a HMT, the histone lysine NH_2_ group undergoes a nucleophilic attack to the methyl group of SAM, producing a methylated lysine and SAH; **b** The overall structure of DOT1L-SAM complex (PDB: 1NW3) and the close-up view of its active site; **c** The overall structure of G9a-SAH complex (PDB: 3K5K) and the close-up view of its active site. SAM/SAH are shown as tube models with their C atoms in green

HMTs belong to a superfamily of methyltransferases containing >100 members from bacteria to humans [9, 10, 18, 19]. In addition to the lysine and arginine sidechains of a protein, methyltransferases can methylate DNA, RNA and even small molecules such as a catecholamine. The methyl acceptors for these enzymes can be a N (e.g., –NH_2_ of a lysine), C (e.g., C5-cytosine in DNA), or O (e.g., –OH of a catecholamine) atom. All methyltransferases use S-adenosylmethionine (SAM) as the enzyme cofactor, with its methyl group (activated by the sulfonium) being the donor. Figure 1a schematically illustrates the general mechanism of catalysis of an HMT. SAM and the substrate histone lysine bind to different binding pockets of HMT in an orientation that brings the methyl donor and acceptor atoms to a close proximity, which facilitates the ensuing nucleophilic substitution reaction to occur, producing the methylated histone and S-adenosylhomocysteine (SAH).

Based on X-ray crystallographic studies, there are five classes of methyltransferases with distinct structural features [9, 10]. It is of interest that H3K79 methyltransferase DOT1L and all PRMTs belong to class I methyltransferases and share high similarities. However, all other HKMTs are class V methyltransferases containing a SET (**S**u(var)3-9, **E**nhancer-of-zeste, **T**rithorax) domain, having a distinct structure from DOT1L. Figure 1b, c show the overall structures as well as the close-up views of the active sites of DOT1L and H3K9 methyltransferase G9a, respectively. DOT1L is a typical class I methyltransferase [20], characterized by an overall protein structure of 7-stranded β sheets flanked by several α helices, as well as an extended binding conformation of SAM (Fig. 1b). The SAM-binding pocket is deeply buried inside the protein. While there has been no structural information as to how nucleosome (the substrate) binds to DOT1L, the substrate-binding pocket is largely separated from that of SAM, interconnected by a narrow lysine binding channel. The class V methyltransferase G9a is a structurally distinct protein, consisting of mostly β sheets interlinked by loops (Fig. 1c) [21]. SAM/SAH adopts a kinked binding conformation in this class of HMTs.

**Fig. 2:**
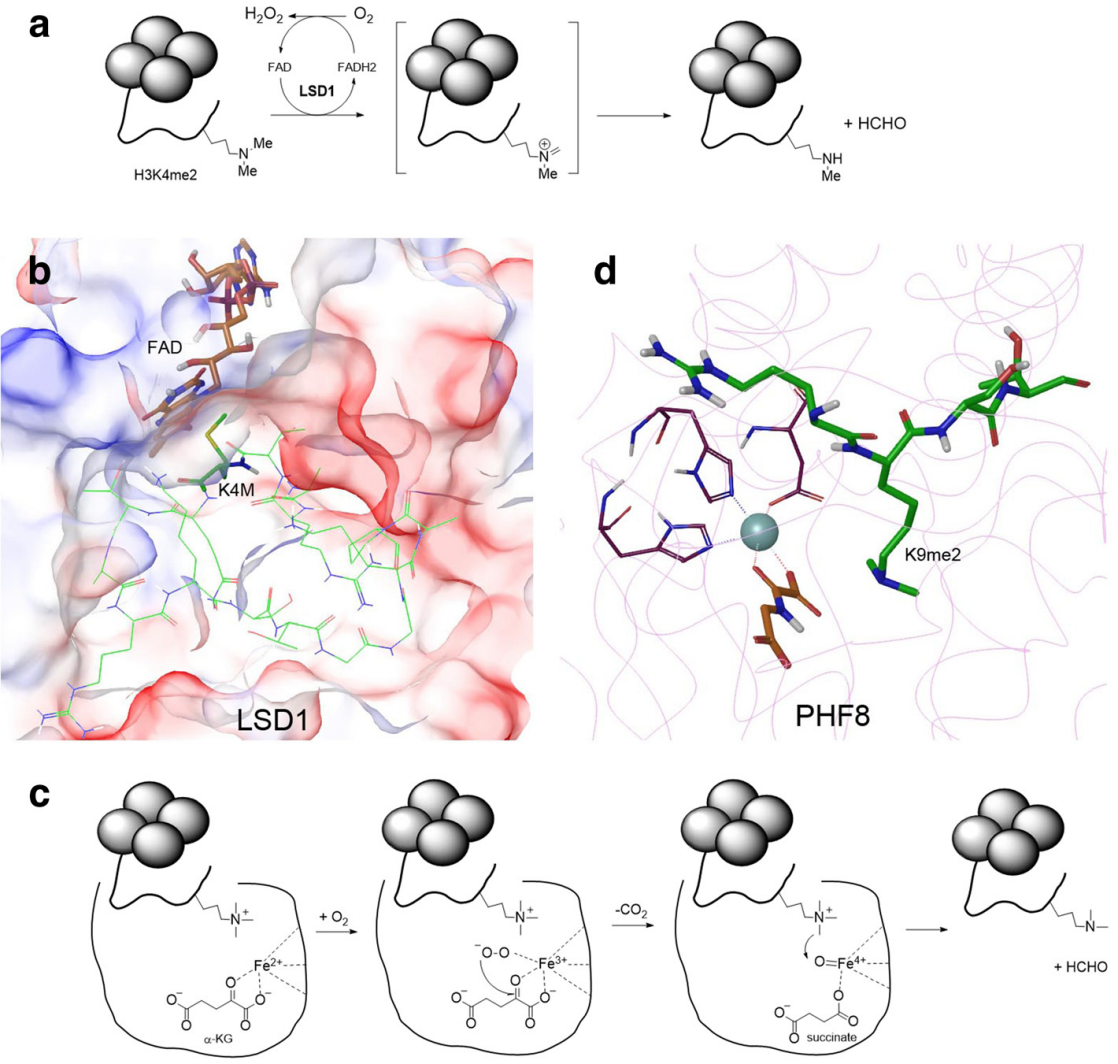
Mechanisms and structures of histone lysine demethylases (KDM). **a** Mechanism of catalysis for FAD dependent KDM1 proteins (including LSD1 and 2); **b** The active site of LSD1 in complex with FAD and a histone H3 peptide (PDB: 2V1D). LSD1 is shown as a 50 % transparent electrostatic surface. The peptide is shown as a wire model (C atoms in *green*), with the K4M residue highlighted as a tube model; **c** Mechanism of catalysis for JmjC domain KDMs; **d** The active site of PHF8 in complex with Fe^2+^ (*cyan sphere*), an α-KG analog (*brown*) and a histone H3 peptide (*green*) (PDB: 3KV4)

For the opposite reaction, there are two families of KDMs that can oxidatively remove the methyl group from a methylated lysine sidechain using distinct mechanisms. LSD1 (also known as KDM1A) and its homolog LSD2 (also known as KDM1B) are flavin adenine dinucleotide (FAD) dependent monoamine oxidases (MAO) [22, 23]. As shown in Fig. 2a, the methyl group of a methylated lysine is oxidized by the cofactor FAD to form an imine intermediate, which hydrolyzes to give the demethylated product and formaldehyde. FADH_2_, the reduced form of the cofactor, is oxidized by O_2_ to generate FAD and H_2_O_2_ to complete a catalytic cycle. Because the formation of an imine intermediate is required, LSD1/2 can only demethylate a mono- or di-methylated lysine, but not a tri-methylated lysine. Figure 2b shows the X-ray crystal structure of LSD1 in complex with FAD and its H3K4 peptide substrate (with K4M mutation) [24]. FAD is tightly bound inside LSD1, with its aromatic tricyclic flavin ring being part of the large substrate-binding pocket, which can accommodate and recognize the histone H3 peptide. The sidechain of Lys4 (mutated to Met) residue is located in proximity to the flavin ring for oxidation.

The other family, consisting of ~30 KDMs including KDM2 - 7 and PHF (plant homeodomain finger) in humans, all contain a JmjC domain and are Fe(II) and α-ketoglutarate (α-KG) dependent dioxygenases [13, 25, 26]. Figure 2c illustrates the general mechanism of catalysis for these enzymes [27, 28]. An oxygen molecule coordinates to Fe(II) and oxidizes both Fe(II) and α-KG to give (upon decarboxylation) a succinate and Fe(IV)-oxo intermediate. Next, the Fe(IV) species oxidizes the C atom of the methylated lysine to form a hydroxymethylamine intermediate, which hydrolyzes to produce the demethylated product and formaldehyde. Unlike LSD1/2, the JmjC family of KDMs can demethylate mono-, di-, and tri-methylated lysine. Figure 2d shows the X-ray structure of PHF8 in complex with Fe^2+^, an α-KG analog and a methylated histone H3 peptide [29]. The central metal ion is coordinated with an Asp and two His residues, together with two O atoms of α-KG. One of the methyl groups of H3K9me2 sidechain is located closely to the metal ion.

**Fig. 3:**
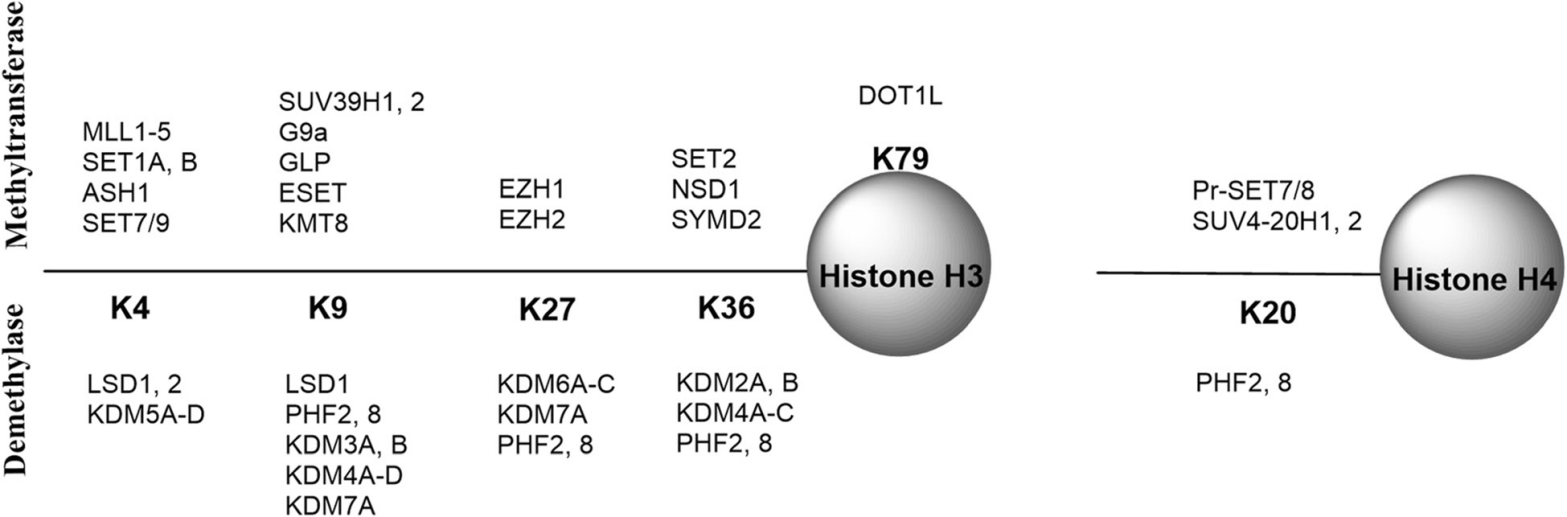
Histone H3 and H4 lysine substrate-specificity of HMTs and KDMs

To date, six lysine residues in histone H3 and H4, i.e., H3K4, K9, K27, K36, K79, and H4K20, have been found to be methylated. Figure 3 illustrates the substrate-specificity of HKMs and KDMs. Many other lysine residues, including H3K14, 18, 23, and H4K5, 8, 12, and 16, are not methylated. Rather, they can be acetylated. It is also of interest that H3K9 and K27 can also be acetylated. Mutually exclusive acetylation or methylation at H3K9 and K27 appears to play drastically distinct physiological functions. Acetylated H3K9 and K27 cause activated gene transcription, while methylated H3K9 and K27 are transcriptional repressive.

## H3K79 methyltransferase DOT1L

### DOT1L’s physiological function and pathogenesis in leukemia

DOT1L (**d**isruptor **o**f **t**elomeric silencing 1 **l**ike) was identified as a human homolog of yeast DOT1, which was found to disrupt telomeric silencing in budding yeast in a genetic screen [30]. The full-length human DOT1L has 1537 amino acids, with its highly conserved N-terminal domain of ~360 amino acids being an H3K79 methyltransferase [31]. The remaining part of mammalian DOT1L is involved in interactions with many transcription proteins, such as AF4, AF9, AF10, and ENL [32–36]. Biology of DOT1L in health and diseases has been summarized in several recent reviews [37–39]. The biological function of DOT1L (as well as DOT1) is to methylate H3K79 as part of a transcription complex, which can initiate or maintain an active transcription state. This is supported by the genetic studies in yeast, showing ~10 % genome containing hypomethylated H3K79 are located at transcriptionally inactive loci, while the remaining 90 % genes with an H3K79 methyl marker are actively transcribed [40, 41]. This also occurs in *Drosophila* and mammals [42, 43]. Several large transcription protein complexes containing DOT1L have been purified and identified using chromatin immunoprecipitation, including ENL-associated proteins (EAP), DOT1L-containing complex (DotCom), and Super elongation complex (SEC) [32–36]. Several transcription relevant proteins were repeatedly present in these complexes, including transcription factors AF4, AF9, AF10, and ENL, as well as P-TEFb kinase. P-TEFb is a cyclin-dependent kinase that can phosphorylates RNA polymerase II, which is required for transcription elongation. These strongly support DOT1L as well as H3K79 methylation is crucial to gene transcription.

DOT1L plays important roles in normal physiology of an organism. For embryonic development, methylation at H3K79 is absent in the very early stage and increasing levels of H3K79me2 can be found in later stages, suggesting this “histone code” is important for embryonic development [44, 45]. Germline knockout of mouse DOT1L was embryonic lethal and major defects in the cardiovascular system were found in the knockout embryos [46]. Additionally, DOT1L has been found to be crucial for maintaining normal hematopoiesis in mice [47, 48]. Conditional knockout of DOT1L in bone marrow significantly decreased hematopoietic stem cells as well as all types of progenitor cells. Moreover, other studies have shown DOT1L plays roles in maintaining normal functions of heart and kidney [46, 49–51].

**Fig. 4:**
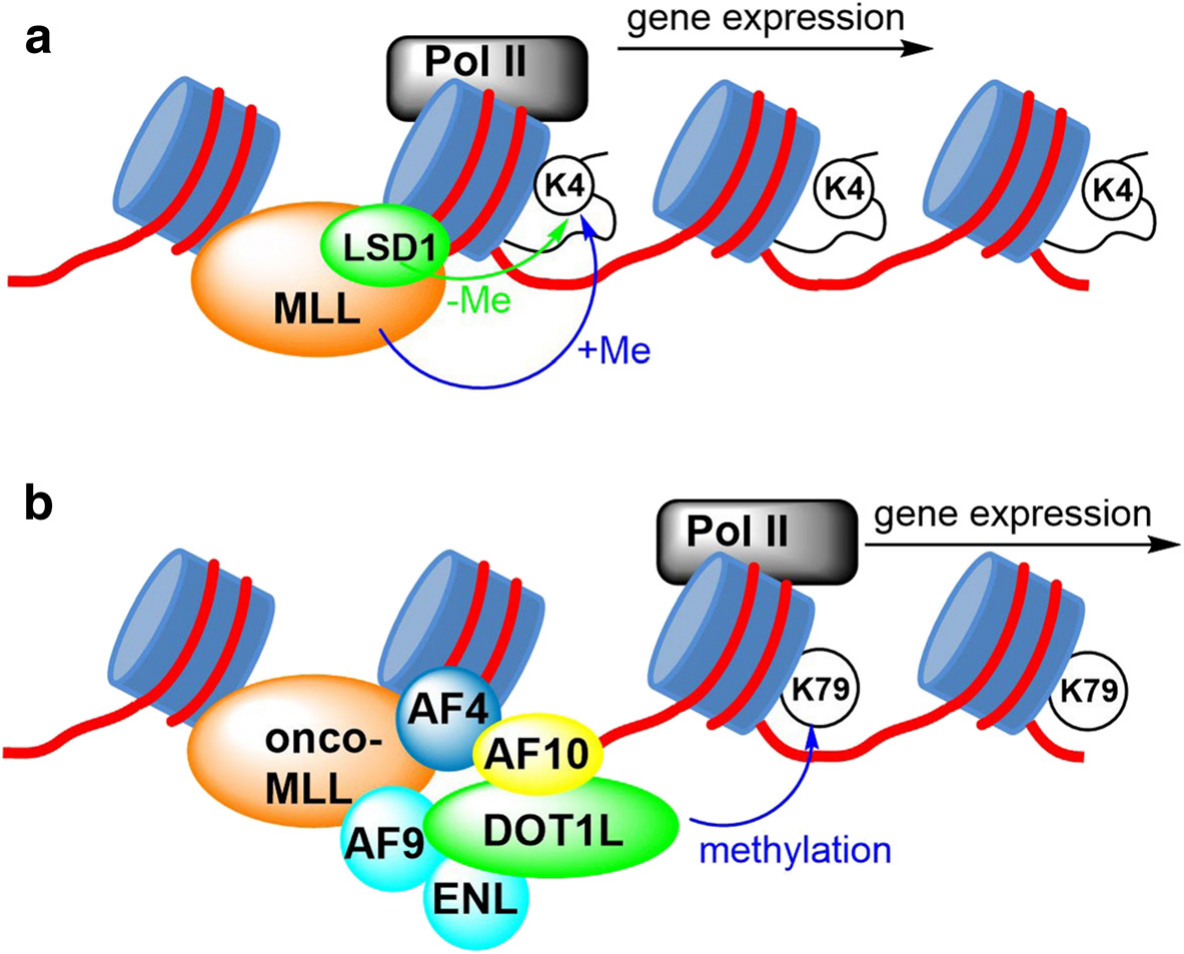
Functions of wild-type MLL, LSD1 and onco-MLL fusion proteins. **a** MLL methylates H3K4 and initiates RNA polymerase II (Pol II) mediated gene transcription, while LSD1 removes the methyl group from H3K4me1 and 2 and keeps a balanced H3K4 methylation; **b** The onco-MLL protein complex involving AF4, AF9, AF10, or ENL can recruit DOT1L, which methylates H3K79 and causes overexpression of leukemia-relevant genes

DOT1L has been found to be a drug target for acute leukemia with a mixed lineage leukemia (MLL, also known as MLL1 or KMT2A) gene translocation. This subtype of leukemia accounts for ~75 % of acute leukemia in infants and ~10 % in children and adults [52–54] with a particularly poor prognosis [55–58]. The phenotype of MLL-rearranged leukemia can be acute myeloid leukemia (AML), acute lymphoid leukemia (ALL), or mixed lineage leukemia. However, despite phenotypic differences, gene profiling showed these MLL-rearranged leukemias share a similar gene expression signature [59]. The biology of MLL and leukemogenesis of MLL-rearranged oncogenes have been well studied and reviewed [60–62]. Briefly, MLL is a large, multi-domain protein (3969 amino acids), containing an N-terminal AT hook domain that recognizes and binds to DNA as well as a C-terminal SET domain that is an H3K4 methyltransferase [52]. Figure 4a schematically illustrates the biology of MLL for gene expression in normal cells. Upon binding to the promoter region of its target genes, the SET domain of MLL can methylate H3K4, which also represents a histone marker for active gene transcription [63, 64]. In the leukemia, the chromosome rearrangement replaces the C-terminal part of MLL with a fusion partner gene [52, 53, 65]. The SET domain as well as its H3K4 methylation activity is thus lost. To date, although >70 partner genes have been documented, onco-MLLs fused with transcription factors AF4, AF9, AF10, and ENL account for the majority (>70 %). As shown in Fig. 4b, these four proteins are able to recruit DOT1L into the MLL transcription complex, which subsequently methylates H3K79 [32, 34, 51, 66, 67]. This aberrant epigenetic event dysregulates the expression of many MLL target genes, such as HoxA9, HoxA7, and Meis1 whose overexpression can cause leukemia. Abnormal H3K79 methylation has been observed in the clinic as well as mouse models of MLL-rearranged leukemia and becomes hallmarks of this malignancy. DOT1L therefore represents a drug target for MLL leukemias. Indeed, this has been established by biological means (e.g., knockdown by RNA interference) [66] and pharmacological inhibition as described below [68–76].

### DOT1L inhibitors and their activity against MLL leukemia

**Fig. 5:**
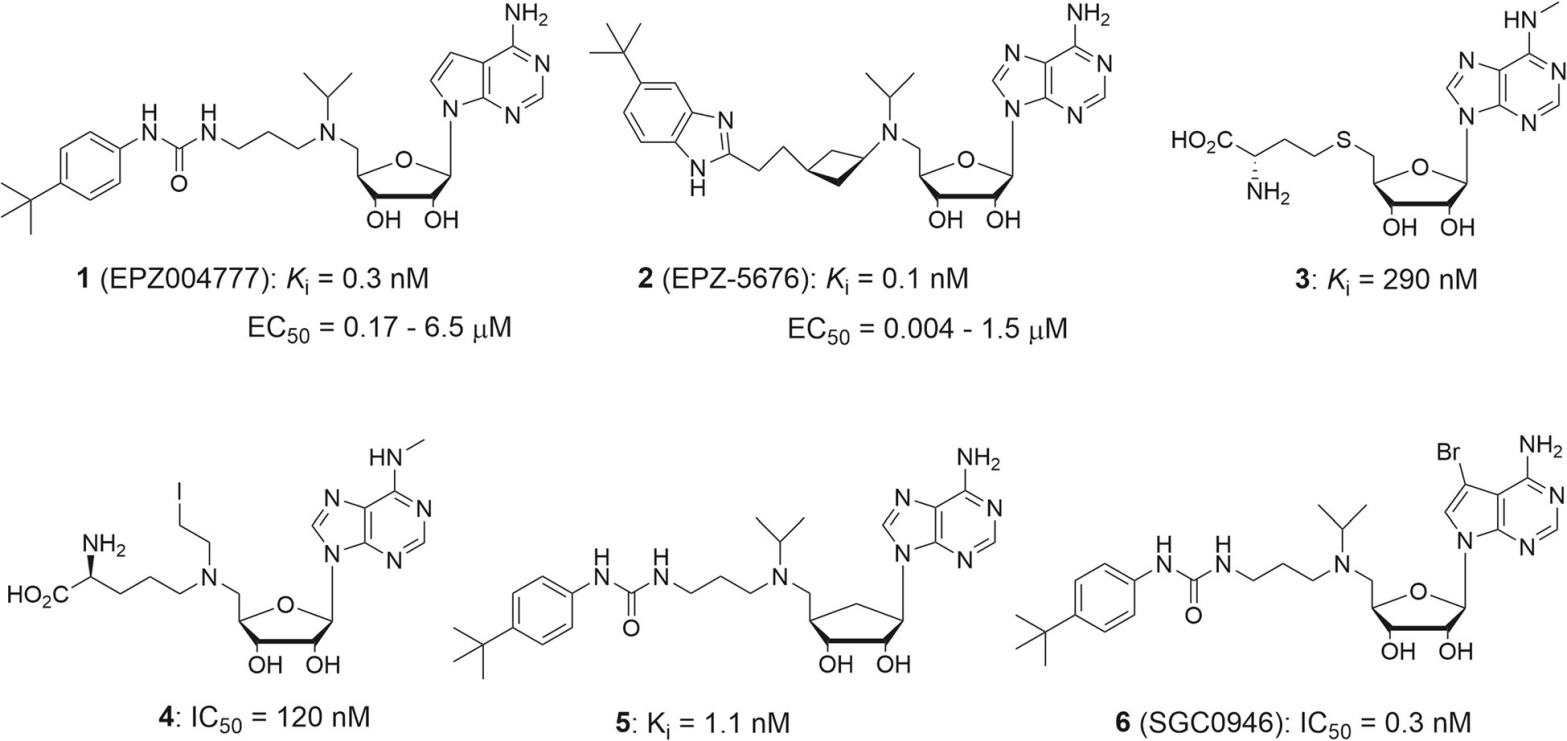
Structures and activities of representative DOT1L inhibitors

Because of DOT1L’s crucial role in oncogenesis and maintenance of MLL-rearranged leukemia, much effort has been dedicated to find small molecule inhibitors of DOT1L. The first DOT1L inhibitor EPZ004777 (1, Fig. 5) as well as its selective antitumor activity against MLL leukemia were reported in 2011 [68]. Several other potent DOT1L inhibitors were disclosed shortly after [69–75]. Figure. 5 summarizes representative inhibitors of DOT1L, as well as their biochemical and antitumor activities. It is noted that all currently disclosed DOT1L inhibitors contain an adenosine or its analogous structure and are competitive to the enzyme cofactor SAM. This is likely because of the difficulty to compete with the substrate nucleosome, which has strong protein-protein and protein-DNA interactions with DOT1L [70].

The discovery of compound 1 followed a conventional ligand-based medicinal chemistry approach [69]. Starting with the natural inhibitor SAH (*K*_i_ = 260 nM), a series of repeated cycles of chemical modifications followed by structure activity relationship studies yielded 1, which still has an adenosine-like moiety, while the sidechain in SAH is replaced with a tert-butylphenyl urea containing tertiary amine group. Compound 1 exhibited an extremely potent inhibitory activity against DOT1L with a *K*_i_ value of 0.3 nM, ~860-fold more active than SAH. In addition, unlike SAH being a broadly active inhibitor of HMTs, 1 shows an excellent enzyme selectivity profile: it did not inhibit a panel of 8 other HMTs at 50 μM, although 1 is a relatively weak inhibitor of PRMT5 (IC_50_: 520 nM). X-ray crystallographic studies showed that the binding of compound 1 causes large protein conformational changes of DOT1L to accommodate the large hydrophobic sidechain [69]. Surface plasmon resonance (SPR) studies revealed that a very slow *k*_off_ rate mostly accounts for the potent inhibitory activity of compound 1. Further inhibitor optimization produced EPZ-5676 (**2**) with an improved activity (*K*_i_ = 0.08 nM) as well as cellular activities [73]. Several other groups also disclosed their DOT1L inhibitors. Structure-based design led to the finding of compound 3, a N6-methyl substituted SAH, which retains the inhibitory activity of SAH against DOT1L and exhibits excellent selectivity against other HMTs [71]. Compound 4 is a potent, mechanism-based inhibitor of DOT1L, with the ability to covalently bind to the substrate [71]. Compounds 5 and 6 are analogs of 1 and also showed highly potent inhibition against DOT1L (*K*_i_ = 1.1 and 0.06 nM) [75, 76]. Compound 5 with a cyclopentane ring (rather than the ribose in other compounds) was designed, synthesized and shown to retain a potent inhibitory activity against DOT1L. However, it exhibited a significantly improved metabolic stability [75]. X-ray structure-based design for compound 6 with a 7-bromo substituent on the adenine ring was intended to exploit a nearby hydrophobic pocket and it turned out that 6 is one of the most potent inhibitors of DOT1L [76].

Compounds 1, 2, 5, and 6 are cell permeable and exhibit selective activities against MLL-rearranged leukemia cells [68, 73, 75]. It is remarkable that these DOT1L inhibitors are not cytotoxic and did not exhibit anti-proliferative activity for a short (e.g., 3 days) treatment. Rather, a long time (e.g., 14 days) incubation is needed, showing a distinct mechanism of action for these compounds [69, 70]. The slow action is likely because of a long time required for a series of cellular events that arrest cell growth, including H3K79 methylation inhibition, followed by systematic changes of gene expression. Compound 1 only inhibited cellular H3K79 methylation with an IC_50_ of ~50 nM and did not affect other histone methylations significantly. 1 inhibited the proliferation of MLL-rearranged leukemia cells with EC_50_ values of 0.17–6.5 μM, while it showed significantly reduced activity against leukemia cells without a MLL rearrangement (EC_50_: 13.9–>50 μM). Treatment with 1 caused downregulation of onco-MLL target genes and induced cell differentiation as well as apoptosis of MLL-rearranged leukemia cells. Gene profiling also revealed that there were significant overlaps in gene expression pattern between samples treated with compound **1** and DOT1L knockdown, supporting DOT1L is the cellular drug target. Due to the poor pharmacokinetics, continuous infusion using an osmotic pump was chosen for administration of compound **1** for in vivo studies. A dose of 70 mg/kg for 21 days was able to cause a regression of subcutaneous tumors of MV4-11 leukemia cells. In a more clinically relevant systemic MV4-11 leukemia mouse model, compound **1** can also prolong survival of the experimental animals with statistical significance.

Compound 2 is the most potent DOT1L inhibitor, together with improved pharmacokinetics [73]. It showed more potent cellular activities (e.g., EC_50_s = 0.004–1.5 μM) and in vivo antitumor efficacy. It has been in phase I clinical trials against MLL-rearranged leukemia. Preliminary clinical results of compound 2 were disclosed in the 56^th^ Annual Meeting of American Society of Hematology [77] as well as several press releases (www.epizyme.com). A total of 37 advanced leukemia patients, who were heavily pretreated with chemotherapies, were enrolled and received 6 doses (ranging from 12 to 90 mg/m^2^/day for 21 or 28 days) of continuous infusion of 2. The compound was well tolerated, with the main adverse events being grade 1 or 2 leukocytosis, nausea and hypomagnesemia. Drug administration can achieve a rapid steady-state plasma concentration of compound 2 on day-1 and cause inhibition of H3K79 methylation in patients’ bone marrow as well as peripheral blood cells. Eight patients out of 34 with an MLL-translocation showed biological or clinical activity, among whom two complete responses as well as one partial response were observed. More clinical trials including combination therapies with other drugs are being conducted.

### DOT1L in other diseases

DOT1L has been identified to be a drug target for several other types of cancers [78–80]. A bioinformatics search found the expression levels of DOT1L correlate with breast cancer as well as a panel of genes that promote proliferation of the malignancy [78]. Knockdown of DOT1L and pharmacological inhibition (by e.g., compound 1) showed inhibition of H3K79 methylation and cell proliferation of several DOT1L^+^ breast cancer cell lines with EC_50_ of 0.19–1.4 μM, while DOT1L^low^ breast cancer cells were not sensitive to DOT1L inhibition. Mechanistically, inhibition of DOT1L/H3K79 methylation can impair self-renewal and metastatic potential, induce differentiation and down-regulate many pro-proliferation genes, all of which contribute to significantly reduced proliferation of these breast cancer cells. H3K79 hypermethylation was observed for lung cancer cell lines A549 and NCI-H1299. siRNA-mediated DOT1L knockdown can block the proliferation of these cells [79]. Therefore, these experiments implied that DOT1L plays an important role in lung cancer. However, no DOT1L inhibitors were tested to confirm this finding. In addition to cancer, DOT1L was recently found to play a role in cell reprogramming. DOT1L knockdown as well as compound 1 were shown to significantly increase the reprogramming efficiency of somatic cells to produce more induced pluripotent stem cells (iPSC), showing the potential of using DOT1L inhibitors in regenerative medicine [80].

## MLL and LSD1 modifying H3K4 methylation

### Cancer biology of MLL and LSD1

The biology of MLL has been extensively studies and reviewed [52–54, 60–62] and briefly summarized in the above section and here. The biological function of MLL is essential for development: knockout of MLL in mice is embryonic lethal. MLL has been found to associate with thousands of gene promoters and have a global role in positive regulation of transcription of many important genes such as Hox families of genes [81, 82]. Hox genes are transcriptional factors essential for the development of multiple tissues including the hematopoietic system, while overexpression of certain members (e.g., HoxA9 etc.) has been found to lead to leukemogenesis [83]. MLL gene translocations are frequently found in acute leukemia. Moreover, it is noted that MLL-translocation occurs in one allele, with the wild-type (WT) MLL in the other allele remaining intact. A recent study showed that MLL’s H3K4 methyltransferase activity is essential for MLL-rearranged leukemia, suggesting inhibition of the SET domain of MLL is a possibly viable approach to MLL leukemia treatment [84].

LSD1 plays an opposite role as a histone lysine demethylase [22, 23]. LSD1 contains four functional domains, including an N-terminal domain with a putative nuclear localization peptide, a SWIRM, and an oxidase domain, inside which there is a tower domain insert [85]. The last three domains are important for demethylation. In addition, the tower domain, which is not present in a closely related enzyme LSD2, directly interacts with CoREST (also known as RCOR1, repressor element-1 silencing transcription factor corepressor 1), through which LSD1 forms protein complexes that regulate histone lysine methylation as well as gene expression. The biological function of LSD1 is crucial, as germline LSD1 knockout in mice was found to be embryonic lethal and conditional knockout caused increased H3K4me1/2, blocked hematopoiesis and pancytopenia (low in all blood cell types) [86]. The primary substrates of LSD1 are H3K4me1 and me2, which are important histone marks for active gene transcription. LSD1 was found to be part of an MLL transcription complex [87]. A possible function of LSD1 is to counteract MLL and keep a balanced H3K4 methylation (Fig. 4a). Of interest is that LSD1 has recently been found to be required for leukemia stem cells transformed with MLL-AF9 [88]. LSD1 knockdown abrogated the transforming ability of MLL-AF9, increased the H3K4me2 levels at MLL-AF9 target gene loci, and reduced the expression of HoxA9 and Meis1. Presumably, LSD1 inhibition could counteract the loss of the SET domain in MLL-AF9 and restore a balanced H3K4 methylation. Pharmacological inhibition of LSD1 showed similar activities against MLL-AF9 leukemia in vitro and in vivo [88]. Although there is a safety concern of LSD1 inhibition [89, 90] because of LSD1’s role in hematopoiesis [86, 91], a recent study showed after termination of LSD1 conditional knockout the impaired hematopoiesis can be recovered in a mouse model [91]. These lines of evidence strongly support that LSD1 is a drug target for MLL leukemia.

In addition, H3K9 and other proteins have been found to be LSD1’s substrates [92–96]. In the context of androgen receptor-mediated gene expression, histone H3 threonine 6 is phosphorylated by PKCβ1, which prevents LSD1 from binding to methylated H3K4. In complex with androgen receptor and PKCβ1, LSD1 can change its substrate-specificity and demethylate H3K9me1 and 2 [92]. DNA methyltransferase 1 (DNMT1), which maintains the integrity of DNA methylation as well as plays an important role in maintaining hematopoietic stem and progenitor cells [97], is also a substrate of LSD1. DNMT1 is methylated in vivo and such methylation destabilizes the protein. LSD1 can demethylate and therefore stabilize DNMT1. Therefore, LSD1 is of importance in maintaining global DNA methylation [93]. In addition, LSD1 can demethylate other non-histone proteins, such as p53 [94], MYPT1 [95], and STAT3 [96], and regulate gene expression mediated by these proteins.

Overexpression of LSD1 has been found in many types of cancer [98–102], including AML (without an MLL-translocation), lung, breast, and prostate cancer. These observations implicate that LSD1 is a potential drug target for these tumors. It was recently found that a significant portion of cell lines of AML and small cell lung cancer (SCLC) are highly sensitive to pharmacological inhibition of LSD1 [103]. Although except for MLL-rearranged leukemia, the molecular mechanisms that link LSD1 to these malignancies are not fully understood (likely because LSD1 has multiple protein substrates), inhibition of LSD1 generally caused broad gene expression pattern changes in these sensitive tumors, which could be responsible for the anti-proliferative activity and other effects, e.g., inducing apoptosis and/or differentiation.

### LSD1 inhibitors and their biological activities

**Fig. 6:**
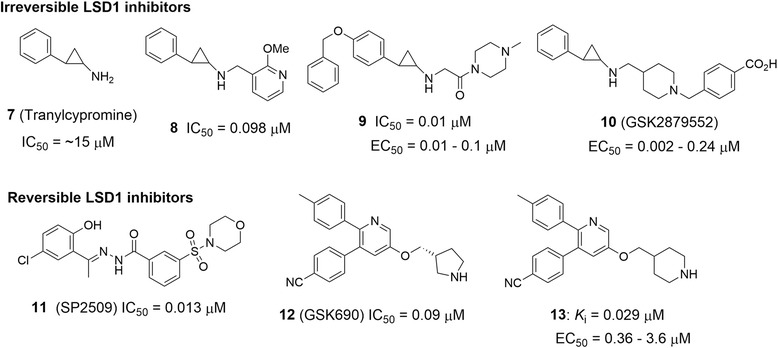
Structures and activities of representative LSD1 inhibitors

A number of small molecule inhibitors of LSD1 have been discovered, developed, reported in the journals and patents [103–112] and reviewed recently [16, 113]. These compounds can be classified into reversible and irreversible inhibitors depending upon their modes of action. We focus on the biological activities of the most potent compounds. Figure 6 summarizes representative inhibitors of these two classes, together with their enzyme and cellular activities. LSD1 belongs to a family of monoamine oxidases (MAO), using FAD as the cofactor for the redox reaction (Fig. 2a). The common feature for irreversible LSD1 inhibitors is that upon oxidation, part of the molecules is able to covalently bind to FAD and permanently deactivates the enzyme [22]. However, for reversible inhibitors, there is no covalent interaction between the inhibitor and the protein.

The first inhibitors of LSD1 with a common cyclopropylamine core structure were derived from Tranylcypromine (7, Fig. 6), an FDA-approved antidepression drug. Compound 7 is an inhibitor of MAO-A and -B, enzymes that degrade neurotransmitters in the central nervous system. Compound 7 weakly inhibits LSD1 with an IC_50_ of ~15 μM. More potent inhibitors have been developed based on the structure of 7. Of importance for the inhibitor optimization is the introduction of a second amine-containing N-substituent, such as those on the right side of cyclopropylamine moiety in highly potent LSD1 inhibitors 8–10. These basic groups not only greatly increase the inhibitory activity, but also render excellent LSD1 selectivity against MAO-A and -B [112]. Compound **8** (compound B in [88]) inhibited recombinant LSD1 in vitro with an IC_50_ of 98 nM. It showed antitumor activities against MLL-AF9 transformed leukemic stem cells. It inhibited colony-forming ability of MLL-AF9 containing leukemia cells with EC_50_ values as low as 50 nM. It also down-regulated expression of many leukemia-relevant genes such as HoxA7, HoxA9 and Meis1. However, it exhibited somewhat severe toxicities in a mouse model of MLL-AF9 leukemia, causing deaths of many experimental mice presumably due to insufficient inhibitory potency and/or inappropriate dosages. Compound 9 (compound 1 in [111]) is a much more potent LSD1 inhibitor, almost quantitatively deactivating the enzyme with an IC_50_ of 9.8 nM. It exhibited potent anti-proliferative activity against MLL-rearranged leukemia cell lines MV4-11 and Molm-13 with EC_50_ values of 10 and 96 nM, while 9 is almost inactive against leukemia cells NB4 and U937 without an MLL-translocation. The differential activities of compound 9 (as well as several other LSD1 inhibitors) suggest that the LSD1 inhibitor is non-cytotoxic, but LSD1 is essential for MLL-rearranged leukemia cells. In vivo antitumor studies using a systemic murine model of MV4-11 leukemia showed that compound 9 did not exhibit overt toxicities and was able to inhibit leukemia progression by >90 % and significantly prolong survival of the experimental animals. Another potent LSD1 inhibitor GSK2879552 (10) was found to exhibit high anti-proliferative activity against 20 out of 29 AML cell lines with EC_50_ values ranging from ~3–100 nM [103]. In addition, anti-proliferation screening of compound 10 led to the finding that a significant portion (9 out of 28) of small cell lung carcinoma (SCLC) cell lines were susceptible to 10 with EC_50_s of ~2–240 nM. This compound also showed significant antitumor activity in a mouse xenograft model of SCLC cancer. Similarly, compound **10** is also devoid of general cytotoxicity: it did not inhibit the growth of >100 cell lines across a range of cancer types, showing a high selectivity of using LSD1 inhibitors targeting cancer. Mechanistic studies showed that gene expression of TGF-β signaling, which is dysregulated in SCLC, was significantly altered upon treatment with compound 10. This could be attributed to the antitumor activity of the LSD1 inhibitor. In addition, DNA hypomethylation of a gene set was identified to be correlated with the sensitivity of SCLC cells (including primary patient samples) to LSD1 inhibition [105]. This biomarker could be used as a major criterion for patient recruitment. Compound 10 has currently been in clinical trials for SCLC, while no clinical data have been disclosed.

Several chemo-types of reversible LSD1 inhibitors have been disclosed, among which compounds SP2509 (11) [107], GSK690 (12) [16], and 13 (compound 17 in [112]) possess low nM in vitro inhibitory activity. Compound 11 potently inhibited LSD1 with an IC_50_ of 13 nM, showing a non-competitive mode of action. Treatment with 11 increased promoter-specific H3K4 methylation, inhibited colony-formation, and induced differentiation and apoptosis of several AML cell lines including MV4-11, Molm-13, and OCI-AML3. The combination of 11 with an inhibitor of HDAC exhibited synergy and showed significantly improved in vivo antileukemia activity in mouse models of AML [107]. Compounds 12 and 13 are quite similar, with the same 3-, 5-, 6-trisubstituted pyridine core structure. Preliminary biological data of compound 12 were presented in the 2013 American Association of Cancer Research annual meeting, showing 90 nM IC_50_ against LSD1, high enzyme selectivity, as well as low μM cellular activity against AML cells. Compound 13 showed an improved in vitro inhibitory activity (29 nM) against LSD1 as well as good anti-proliferative activities (EC_50_: 0.36–3.6 μM) against several sensitive cancer cells including MV4-11 with MLL-AF4 oncogene [112].

### MLL inhibitors

**Fig. 7:**
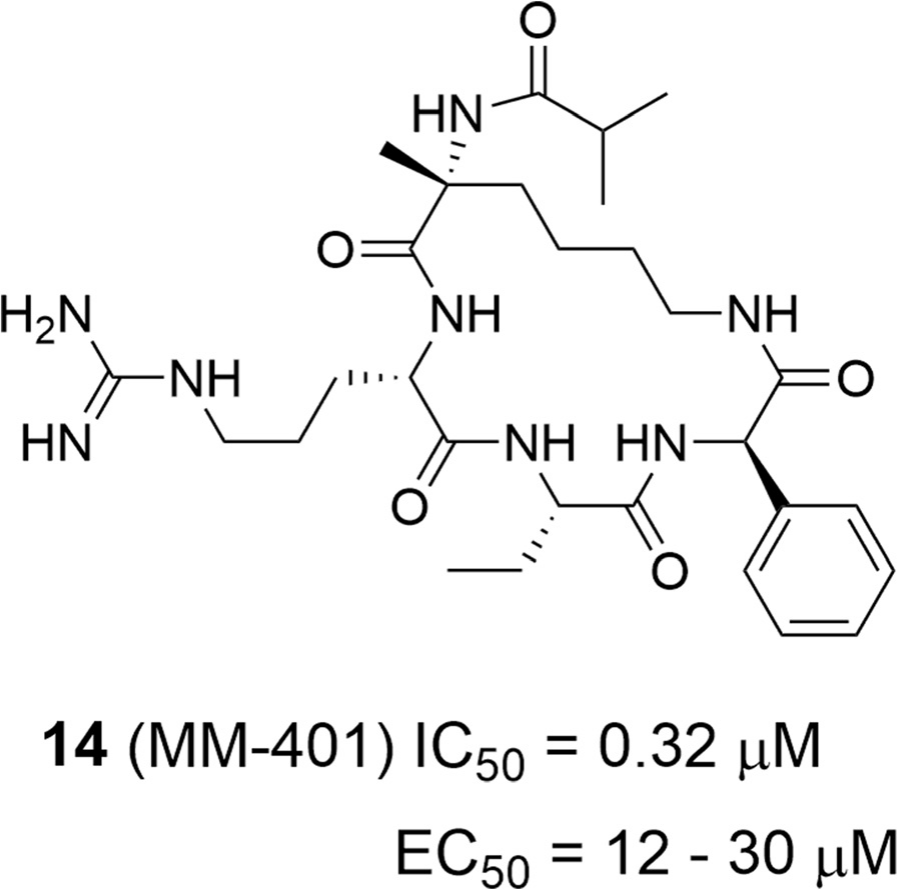
Structure and activity of a compound that disrupts MLL:WDR5 interactions and thereby inhibits MLL indirectly

In MLL-rearranged leukemia, the MLL gene translocation is heterozygous. The H3K4 methyltransferase activity of the remaining copy of WT MLL was found to be essential for the malignancy [92]. Therefore, MLL inhibitors could be useful to treat MLL-rearranged leukemia. However, compounds that directly inhibit the SET domain of MLL have not been reported. Alternatively, the MLL SET domain alone was found to have extremely low methyltransferase activity [114, 115]. The optimal enzyme activity requires its complexation with three other proteins, i.e., WDR5, ASH2L, and RbBP5. Among these, the interaction between WDR5 and MLL is critical, which led to the finding of indirect MLL inhibitors, compounds that disrupt the binding of WDR5 to MLL [116, 117]. Several compounds have recently been found to bind to WDR5 with *K*_d_ values of <0.001–5.5 μM. To date, the best compound MM-401 (14, Fig. 7), an extremely tight binder to WDR5 with a *K*_d_ value of <1 nM, showed an IC_50_ of 0.9 nM in disrupting the interaction between WDR5 and MLL. Indeed, compound 14 almost quantitatively inhibited the methyltransferase activity of the MLL complex (0.5 μM) with an IC_50_ value of 0.32 μM in vitro. Because the WDR5-MLL interaction is unique, compound 14 exhibited a high enzyme selectivity profile: it did not inhibit several closely related SET domain methyltransferases including MLL2 - 4, SET1, and SET7/9. Consistent with MLL’s role, it showed selective activity against MLL-rearranged leukemia cells. Compound 14 inhibited the proliferation of MLL-rearranged leukemia cells with EC_50_ values of 12–30 μM, while it had no effects on other non-MLL leukemia cells. The relatively weak cellular activity might be due to the poor cell permeability of compound 14, a cyclic peptidomimetic compound.

## H3K27 methyltransferase EZH2

### H3K27 methylation and EZH2 in health and cancer

SET domain containing EZH2 (Enhancer-of-zeste homolog 2) and its close homolog EZH1 catalyze mono-, di- and tri-methylation of H3K27 [118–120]. EZH2 was originally identified as a member protein of the Polycomb Repressive Complex 2 (PRC2), which functions as a transcription repressor of Hox gene clusters important to the development [121–123]. In 2002, it was further determined to be a methyltransferase of H3K27 [118–120]. EZH2 alone is not catalytically active. Complexation with EED and SUZ12, two other members in PRC2, is required to methylate H3K27 [124, 125]. The biology of EZH2-containing PRC2 as well as their mediated H3K27 methylation has been reviewed [121–123, 126]. Briefly, EZH2 is essential for embryonic development and plays important roles in normal physiology. EZH2 mediated H3K27me3 has been found to be a “histone code” for transcription repression, while the genes silenced by EZH2/H3K27me3 are different, depending upon cell types [127]. Such epigenetic transcription repression is essential to cell fate determination and function. However, the underlying regulatory mechanism for the gene silencing is complicated and not fully understood. Other proteins in the PRC2 complex also contribute to the gene silencing. For example, studies showed that DNMT is associated with EZH2 in PRC2 and EZH2 is required to recruit DNMT to its target gene promoters and methylate the DNA [128]. This finding showed the two epigenetic events, i.e., H3K27me3 and DNA methylation, are interconnected for transcription repression. In addition, although the homologous EZH1 also catalyzes H3K27 methylation as well as associates with PRC2, it has different biological functions and cannot substitute EZH2 [129].

Overexpression of EZH2 is often found in a variety of cancers such as breast, prostate, lung and blood cancers [130–135], with its expression level correlated with that of H3K27me3, more virulent progression of the disease, and poor prognosis. Further studies suggested that EZH2 plays important roles in tumorigenesis, progression as well as metastasis [136, 137]. However, it remains to be answered in these cases whether the methyltransferase activity of EZH2 is required for these cancers. Using potent and selective EZH2 inhibitors, studies have recently shown that proliferation of several types of cancer cells was not significantly affected by pharmacological inhibition of EZH2/H3K27 methylation [78, 138, 139].

Recently, somatic mutations of EZH2 at Y641, and A677 were identified in 12–25 % non-Hodgkin lymphomas [140–142]. Biochemical investigation revealed that these EHZ2 mutants exhibit a different substrate-specificity from that of the WT enzyme [143–145]. The WT EZH2 catalyzes the mono-methylation of H3K27 with the highest speed, while it is significantly less efficient for the second and especially third methylation of H3K27. A reversed trend was observed for the Y641 mutant proteins: they are inactive for the first methylation, but these mutants transfer the second and third methyl to H3K27 in an increasing catalytic efficiency. In addition, the A677G mutant EZH2 can catalyze all three methylation at a high speed. Clinically, these EZH2 mutated lymphomas exhibit a particularly high levels of H3K27me3.

### EZH2 inhibitors

**Fig. 8:**
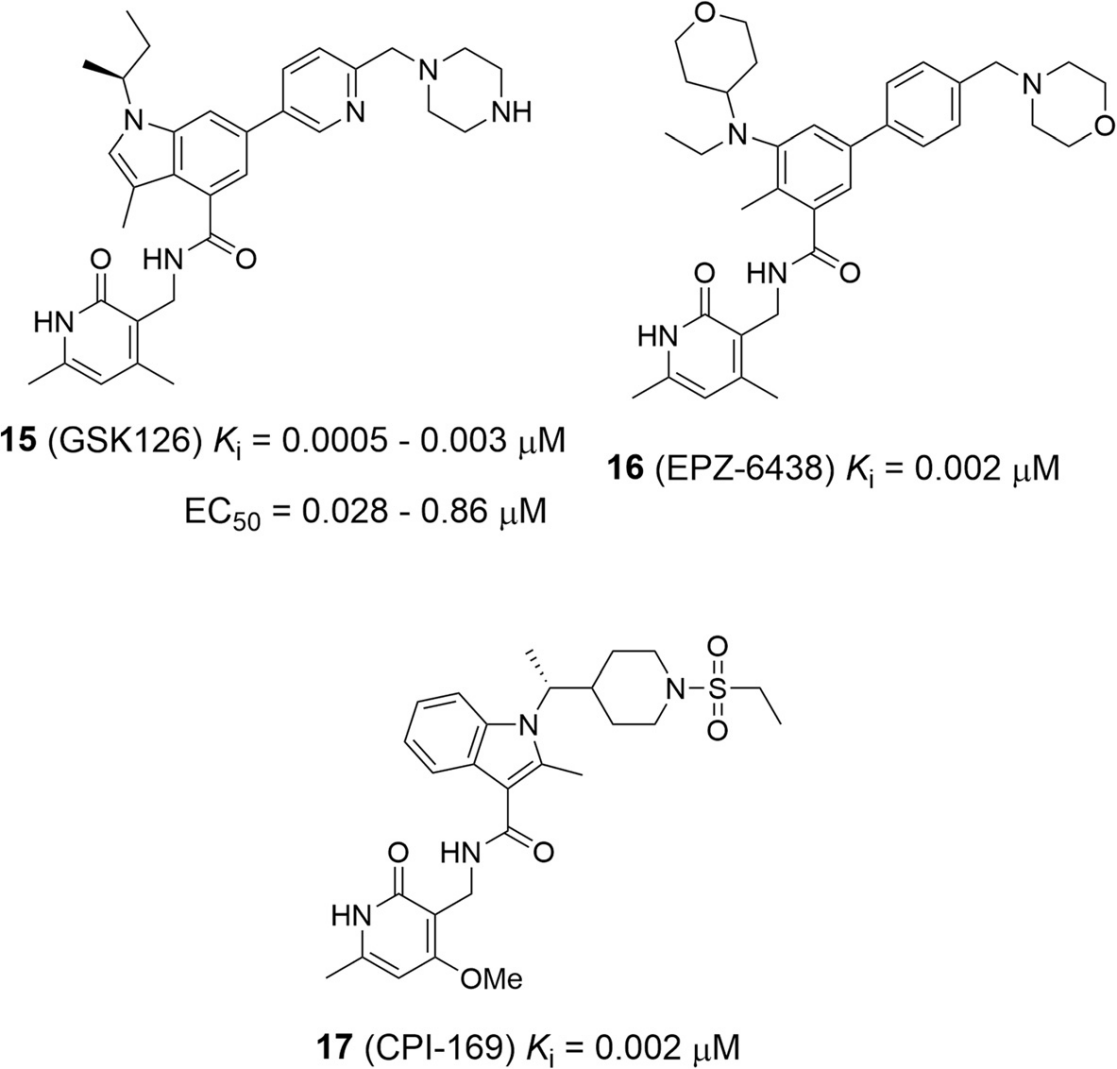
Structures and activities of representative EZH2 inhibitors

Recent high-throughput screening campaigns followed by medicinal chemistry have identified several potent and highly selective inhibitors of EZH2, as representatively shown in Fig. 8. GSK126 (15) is the first disclosed inhibitor with a *K*_i_ value of 0.5–3 nM against WT and all mutant EZH2 enzymes [139]. It showed >150-fold selectivity against EZH1 (*K*_i_ = 89 nM), as well as >1000-fold selectivity against other HMTs. Enzyme kinetics studies suggested that compound 15 is competitive with the enzyme cofactor SAM and non-competitive with the substrate H3K27. It can potently inhibit cellular H3K27me3 levels with IC_50_ values of 7–252 nM in a broad variety of cell lines, independent upon the status of EZH2 mutation. Compound 15 was found to have strong anti-proliferative activities (EC_50_ = 28–861 nM) against diffuse large B-cell lymphoma cells containing an EZH2 activating mutation, while lymphoma cells without an EZH2 mutation are generally insensitive. The antitumor activity has also been confirmed in a mouse model study. Treatment with compound 15 was able to cause the xenograft tumors to regress in a dose-dependent manner and significantly prolong the survivals of the tumor bearing mice. Mechanistically, treatment with compound 15 reduced the cellular H3K27me3 levels and caused a broad range of transcriptional activation of EZH2/PRC2 target genes in sensitive lymphoma cells, while it did not significantly affect the gene expression patterns of insensitive cells.

Scientists from several other institutes have also reported their potent EZH2 inhibitors with enzyme IC_50_ values in the very low nM range [138, 146–149], as shown representatively in Fig. 8. Most of these potent EZH2 inhibitors contain the same or similar N-(2-pyridinone-3-yl)methyl amide substructure, suggesting this moiety is important for protein binding. Enzyme kinetics studies showed that these compounds exhibited a competitive mode of action against the cofactor SAM, suggesting that these compounds bind to the SAM-binding site in EZH2. However, no X-ray structures of EZH2-inhibitor complexes have been reported to date. The exact binding modes of these compounds still need to be explored, which could provide useful structural information for further rational inhibitor design targeting EZH2. Recently, EZH2 inhibitors 16 and CPI-1205 (with an undisclosed structure) have entered clinical trials against EZH2 mutated non-Hodgkin lymphomas, while the results have not been disclosed.

## Isocitrate dehydrogenase (IDH) mutation and histone methylation

### IDH mutation in glioma and other cancers

Isocitrate dehydrogenase (IDH) is one of the key enzymes in the tricarboxylic acid cycle for aerobic metabolism of glucose [150]. There are three IDH isozymes in humans, with IDH1 located in cytoplasm and IDH2 and 3 in mitochondria [151]. Recent genetic studies have identified recurrent mutations of IDH in a number of cancers. IDH1 mutations were found in ~75 % low-grade gliomas as well as secondary glioblastoma multiforme (GBM), the grade IV glioma developed from the low-grade tumors [152–156]. Of particular interest is that all these mutations occur exclusively in the R132 residue of IDH1 or the corresponding R172 residue of IDH2, with the R132H mutation being predominant (>90 %) in these gliomas. Mutations of IDH1 or 2 have also been found in ~20 % AML and several subtypes of sarcomas [157–160]. The IDH mutations occur at an early stage of these cancers, suggesting they could play an important role in oncogenesis [161–163].

**Fig. 9:**
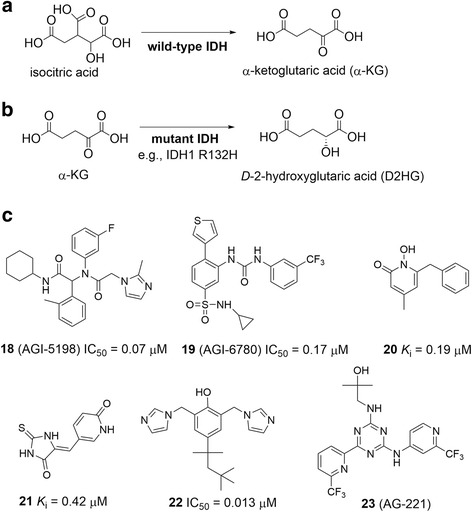
Mechanisms and inhibitors of IDH. **a** Mechanism of catalysis for the wild-type IDH. **b** Mechanism of catalysis for mutant IDHs. **c** Structures and activities of representative inhibitors of mutant IDH

Biochemically, although mutant and WT IDH enzymes do not directly work on histones, the products of their catalyzed reactions are an inhibitor or cofactor of JmjC domain histone demethylases (Fig. 2c) and therefore affect histone lysine methylation. As shown in Fig. 9a, WT IDH catalyzes the oxidative decarboxylation of isocitric acid to produce α-ketoglutaric acid (α-KG) [150], while mutant IDH enzymes lose this function [164, 165]. Rather, all these mutants catalyze a new reaction, the reduction of α-KG to *D*-2-hydroxyglutaric acid (D2HG) (Fig. 9b) [157, 164, 166]. This new function generates >100-fold elevated concentrations of D2HG in tumor cells bearing an IDH1 or IDH2 mutation, while there is only trace amount of D2HG in normal cells. The extremely high level of D2HG is therefore a hallmark of IDH mutated glioma and is suggested to be used as a biomarker for these types of cancers [157, 164, 166]. D2HG with its structure being very similar to α-KG has been found to inhibit α-KG-dependent KDMs and TET family of 5-methylcytosine hydroxylases [159, 167], which are important enzymes to maintain a balanced methylation status of histone and DNA. IDH mutated cancers exhibited genome-wide hypermethylation of histone and DNA [159, 167–169]. In addition, transfection of IDH1 R132H mutation into U87 GBM cells without an IDH mutation blocked cell differentiation and also caused DNA/histone hypermethylation in a very similar fashion to that observed in the clinical tumor samples [168, 170]. These lines of evidence strongly support IDH mutation is a drug target for intervention.

## Inhibitors of mutant IDH

Significant efforts from the academia and pharmaceutical industry have been given to discover small molecule inhibitors of mutant IDH. A number of potent and selective inhibitors of mutant IDH1 and 2 with several chemo-types have been published [171–177] and Fig. 9c summarizes several representative compounds together with their biological activities.

AGI-5198 (18) and AGI-6780 (19) with distinct chemical structures were the first inhibitors of mutant IDH1 and IDH2, respectively [172, 173]. Dipeptide-like compound 18 exhibited potent inhibitory activity against IDH1 R132H (IC_50_ = 70 nM) and R132C (IC_50_ = 160 nM) mutants, respectively, while it did not inhibit WT IDH1 or WT and mutant IDH2 enzymes (IC_50_ > 100 μM). This compound can also reduce cellular D2HG concentrations and the colony-forming ability of glioma cells with IDH1 R132H mutation. Because of the reduced cellular D2HG as well as restored activity of histone demethylases, H3K9 methylation, a repressive histone mark, was found to be decreased globally as well as in several gene promoters. Compound 18 exhibited in vivo antitumor activity in a mouse xenograft model of IDH1 mutated glioma. Compound 19 is a urea-substituted benzenesulfamide compound, showing selective activity against various mutant forms of IDH2 with IC_50_ values of 4 – 170 nM, while it exhibited high selectivity against WT IDH2 or IDH1 enzymes. It can similarly reduce cellular D2HG concentrations effectively, inhibit proliferation and colony-forming ability, and promote differentiation of primary AML cells harboring an IDH2 mutation. Compounds 20–22 were reported as potent and selective inhibitors of mutant IDH1, showing 0.013–0.42 μM inhibitory activities [174–177]. Compounds 21 and 22 can also selectively impair colony-forming ability of patient derived glioma cells having R132H IDH1 mutation.

AG-221 (23), an advanced inhibitor of mutant IDH2, has been in phase I clinical trials against AML or myelodysplastic syndrome with IDH2 mutations. Based on data disclosed in the company’s press releases (www.agios.com), compound 23 exhibited promising clinical pharmacokinetic, safety and efficacy profiles. A total of 177 patients, who have relapsed or refractory AML with IDH2 mutations, have been treated with various doses of compound 23. The drug seems to be well tolerated, with the majority of adverse effects being mild to moderate, including nausea, fatigue, increased blood bilirubin and diarrhea. The maximum tolerated dose of 23 has not been reached. Preliminary pharmacokinetic and pharmacodynamics studies showed compound 23 has an excellent oral availability, and a high plasma half-life of >40 h. Objective responses have been observed for 63 patients out of 158 evaluable patients, showing an overall response rate of 40 %. Among these, there were 26 cases of complete remission (CR), three cases of CR with incomplete platelet recovery, 14 cases of marrow CR, two cases of CR with incomplete hematological recovery as well as 18 cases of partial remission. Given these promising clinical data, more clinical evaluations of compound 23 as well as several other inhibitors of mutant IDH are currently on-going worldwide.

## Inhibitors of selected other histone methylation enzymes

**Fig. 10:**
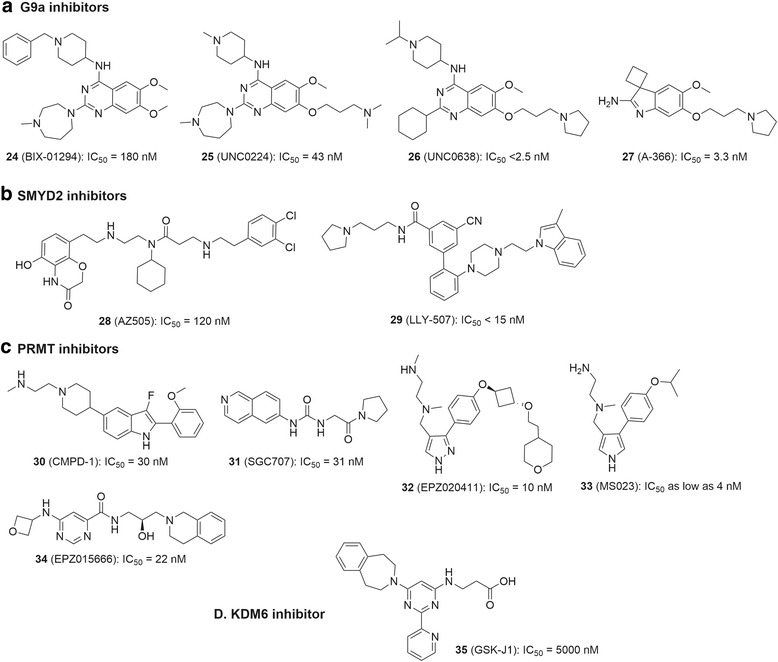
Structures and activities of representative inhibitors of **a** G9a/GLP; **b** SMYD2; **c** PRMTs; and **d** KDM6

There are excellent review articles that summarize the biology of HKMTs, PRMTs and KDMs as well as their relevance to cancer or other diseases [4, 8, 15–17, 178]. As compared to the large number (>100) of these histone methylation enzymes, relatively fewer number of potent and selective small molecule inhibitors have been discovered in the past few years. We describe below potent inhibitors of several other important histone methylation modifying enzymes (Fig. 10). Most of these compounds are cell permeable and can be used as chemical probes for biological studies of these proteins. However, a number of previously reported inhibitors are not included, because of low potency (e.g., with μM enzyme IC_50_s), non-specificity, or unknown mechanism of action. It is therefore unclear whether the observed biological activities of these compounds are due to inhibition of the protein target or off-target effects.

### Inhibitors of other HKMTs

G9a (also known as EHMT2) and its closely homologous protein GLP (also known as EHMT1) are responsible for mono- and di-methylation of H3K9 [179] and play important roles in gene regulation. There is also strong evidence that G9a/GLP is implicated in several types of cancers [180–182]. The first specific inhibitor of G9a is BIX-01294 (24, Fig. 10a) identified in a high-throughput screening [183]. Compound 24 exhibits an IC_50_ of 180 nM against G9α, whereas no inhibition of the other histone methyltransferases, such as SUV39H1 and PRMT1, was observed. In a cellular assay, 24 was found to transiently modulate H3K9me2 marks. Medicinal chemistry modifications based on the scaffold of 24 yielded a series of compounds with improved potency as well as cell permeability, such as UNC0224 (25) [21] and UNC0638 (26) [184]. Compound 25 exhibited an improved IC_50_ of 43 nM against G9α by adding a lysine mimetic group. An X-ray crystal structure of G9a in complex with 25 revealed that this group occupies the lysine binding channel of the protein. Compound 26 is a potent, selective and cell permeable G9a inhibitor, able to reduce H3K9me2 levels in a dose-dependent manner with an EC_50_ of 81 nM. Furthermore, compound 26 can reduce colony-forming ability of breast cancer MCF-7 and MDA-MB231 cells. Compound A-366 (27) with a distinct chemo-type is also a highly potent and selective inhibitor of G9a with an IC_50_ of 3.3 nM [185].

SMYD2 is a SET domain-containing methyltransferase with a less restricted substrate-specificity. H3K4 and H3K36 as well as non-histone proteins such as HSP90 and p53 were found to be a substrate of SMYD2. Because of the observed overexpression in a variety of cancer types [186, 187], it is implicated as a drug target. The first SMYD2 specific inhibitor AZ505 (28, Fig. 10b) was identified in a high-throughput screening, showing an enzyme IC_50_ of 120 nM as well as selectivity indices of >690-fold [188]. The steady-state kinetic studies indicated that this compound is competitive to the peptide substrate and uncompetitive for the cofactor SAM, which was confirmed by an X-ray crystallographic investigation of the SMYD2-28 complex. LLY-507 (29) was disclosed to be a more potent inhibitor of SMYD2 (IC_50_ < 15 nM), together with >100-fold selectivity over other KMTs [189]. Compound 29 inhibited cellular methylation with an EC_50_ of ~600 nM, showing it is a good chemical probe for investigation of the functions of SMYD2 in normal physiology and diseases.

### Inhibitors of PRMTs

There are two types of PRMTs [9]. The majority of PRMTs (including PRMT1-4, 6, and 8) are the type I enzymes, catalyzing mono- and asymmetric di-methylation of an arginine sidechain. PRMT5 is the only known type II enzyme, catalyzing symmetric di-methylation of an arginine.

CMPD-1 (30, Fig. 10c) is a potent inhibitor of PRMT4 (also known as CARM1) with an enzyme IC_50_ of 30 nM as well as selectivity indices of >300-fold against PRMT1 and 3 [190]. X-ray crystallography showed that compound 30 occupies the substrate-binding pocket and the high selectivity arises from sequence differences between PRMT4 and PRMT1/3.

An allosteric inhibitor SGC707 (31) is a highly potent inhibitor of PRMT3 an IC_50_ of 31 nM [191]. It exhibited no inhibitory activity against a panel of 27 protein methyltransferases. Crystallographic studies of the PRMT3-31 complex revealed that the inhibitor occupies a cavity >15 Å from the enzyme active site. In cellular assay, compound 31 was able to reduce H4R3me2 with an EC_50_ of 225 nM.

EPZ020411 (32) has been reported to inhibit PRMT6 with an IC_50_ of 10 nM, while it exhibited good selectivity (>10 fold) against PRMT1 and 8 [192]. In cellular assays, treatment with 32 resulted in a dose-dependent decrease of H3R2me2 with an EC_50_ of 640 nM.

Besides the above selective inhibitors against an individual PRMT enzyme, it is of interest that MS023 (33) is a pan-inhibitor of type I PRMTs [193]. It was designed based on the structures of PRMT4 inhibitor 30 and PRMT6 inhibitor 32. Compound 33 exhibited an enzyme IC_50_ of 30, 119, 83, 4, and 5 nM against PRMT1, 3, 4, 6, and 8, respectively. Treatment with 33 significantly decreased levels of H3R2me2 and H4R3me2 with EC_50_s of 56 and 9 nM, respectively.

EPZ015666 (34) was reported as a potent and selective inhibitor of PRMT5 (IC_50_ = 22 nM), a type II PRMT [194]. Compound 34 demonstrated potent anti-proliferative activity against mantle cell lymphoma cells Z-138 and Maver-1 (EC_50_ = 96 and 450 nM, respectively), because of overexpression of PRMT5 in this type of tumor. It also showed in vivo antitumor activity in xenograft mouse models of these two lymphoma cells.

### Inhibitors of other KDMs

Despite a number of compounds were reported to be inhibitors of the JmjC family of KDMs, they are either analogs of α-KG or metal chelators (e.g., hydroxamates and pyridinyl carboxylates) [16]. These compounds generally lack potent inhibitory activity (mostly having μM IC_50_s) and enzyme selectivity. In addition, cellular activities, such as histone methylation changes, anti-proliferation and/or toxicity, of these compounds have not been well investigated. Recently, GSK-J1 (35) was reported to be a selective inhibitor of KDM6, responsible for demethylation of H3K27. Compound 35 was initially identified as a lead in a high-throughput, fluorescence based AlphaScreen assay and later confirmed to selectively inhibit KDM6B with an IC_50_ of 5 μM, while it exhibited ~10-fold selectivity against KDM4 enzymes [195]. X-ray crystallography showed 35 binds to the active site of the protein with two of its N atoms chelating the metal ion. Compound 35 is not cell permeable, while its ester prodrug GSK-J4 was found to inhibit cellular production of TNF-α with an EC_50_ of 9 μM. While the global histone methylation changes were not determined, GSK-J4 inhibited a reduction of the H3K27me3 level at the *TNFA* gene promoter using chromatin immunoprecipitation (ChIP) assays, suggesting it is on-target in cells.

## Perspectives and conclusions

Histone methylation plays critical roles in gene regulation, cell differentiation, DNA recombination and damage repair. Histone methylation modifying enzymes, which are often part of a transcription complex, have been heavily studied for the past few decades and great amount of understanding has been obtained for their biochemistry, structures and biological functions in normal physiology as well as in pathogenesis. Many of these enzymes have been validated or implicated as drug targets for intervention. Small molecule inhibitors of these proteins are not only potential therapeutics, but also useful chemical probes for investigation of biological functions of histone modifying enzymes. To date, discovery and development of small molecule inhibitors of HMTs and KDMs have been still in a very early stage, with significant efforts and successful stories from the academia and pharmaceutical industry occurring only in the past few years. However, it is exciting that the field has been growing exponentially: as described in this review, highly potent and selective inhibitors of several such proteins, including DOT1L, LSD1, EZH2, and mutant IDH, have been developed and undergone extensive preclinical testing. Several compounds have quickly entered and been evaluated in clinical trials in just a few years. They have demonstrated promising clinical safety and efficacy, showing great potentials to become clinically useful drugs. These remarkable outcomes could further attract more scientists to the field and jump-start drug discovery and development endeavors targeting histone methylation for cancer therapy. However, due to important functions of histone methylation in normal physiology, inhibition of histone methylation enzymes could cause toxicities. Vigorous toxicological studies should be performed to ensure there is a sufficient therapeutic window for such pharmacological inhibition.

Additionally, small molecule compounds targeting histone methylation enzymes could provide great opportunities for finding new cancer biology. Given the differences between pharmacological inhibition and genetic knockdown, using these compounds as probes can complement biological means to explore the functions of a histone methylation protein in health and diseases. For example, many of these proteins (e.g., MLL and DOT1L) contains multiple domains having more functions than being a methyltransferase. Pharmacological inhibition of the catalytic domain of the protein, without disturbing the functions of other domains, could provide more useful insight. Moreover, in the context of cancer therapy, overexpression of a certain histone methylation protein is often found in cancer. However, it is still unclear whether the cancer is dependent upon the catalytic activity of such protein. These compounds can be used to validate whether the protein is a real drug target. Many of the inhibitors shown in Figs. 4, 5, 6, 7, 8, 9, and 10 are commercially available for these studies. The availability and costs can be found in PubChem (https://pubchem.ncbi.nlm.nih.gov/).

There are still a number of challenges in the field from the perspectives of chemistry and biology. From the chemistry side, finding potent and selective small molecule inhibitors is a major challenge, because these enzymes share a relatively high homology and use the same cofactor. This requires an enormous amount of coordinated efforts in high-throughput screening, ligand and structure-based inhibitor design and synthesis, medicinal chemistry as well as biological activity testing. If successful, this is, however, the first step towards yielding a clinically useful drug. Repeated rounds of medicinal chemistry, in vitro and in vivo pharmacokinetics, toxicity and efficacy testing are needed to find suitable drug candidates, which is followed by a low success rate in clinical trials. Very high costs for the whole process represent another barrier. From the biology side, more studies need to be performed to validate whether a histone modifying enzyme is a cancer target. Because of the high costs for high-throughput screening and medicinal chemistry, it is desirable that sufficient evidence showing a critical function of the enzyme has been found in the disease before compound development. Genetic screening of gene mutations in clinical tumor samples represents a powerful means to identify potential candidates (e.g., work in 5.1 and 6.1 for mutations of EZH2 and IDH and [196]). Ensuing biochemical and biological studies are needed to further identify the links between the functions of such mutation and pathogenesis. In addition, determination and comparative analysis of methylation levels at different histone residues (e.g., H3K79me2 in [67]) between normal and patient samples could provide useful hints. If there are available resources, direct screening a collection of specific inhibitors of histone modifying enzymes against a large number of cancer cell lines is also a fruitful measure to find such links (e.g., work in [103]). With the successful stories summarized here, it is envisioned that more histone methylation enzymes can be identified as therapeutic targets for cancer.

Combination therapy is a viable approach to increase the effectiveness of a histone methylation modulator. Because multiple histone modifications that change global gene expression might be involved in a certain type of cancer, combination therapy targeting two or more closely associated epigenetic changes could be synergistic. For example, strong synergy was observed for combination treatment using inhibitors of LSD1 and DOT1L that closely associated in MLL-rearranged leukemia [111]. Synergy was also found for combination therapy of a LSD1 inhibitor with an HDAC inhibitor in vitro and in AML mouse models [107]. Aberrant methylation of DNA and histone are often associated [197–199], combination with a DNA methylation inhibitor could also be useful. To rationally design a synergistic combination therapy, mechanistic studies of oncogenesis at the molecular level are helpful to find two possible candidate proteins for intervention [200]. In addition, combination therapy is useful to overcome drug resistance as well as reduce toxicities and side effects.

## Abbreviations

AML, acute myeloid leukemia; ALL, acute lymphocytic leukemia; DOT1L, disruptor of telomeric silencing 1 like; D2HG, D-2-hydroxyglutaric acid; DNMT, DNA methyltransferase; EZH2, Enhancer-of-zeste homolog 2; FAD, flavin adenine dinucleotide; H3K4, histone3-lysine4; H3K79, histone3-lysine79; HDAC, histone deacetylases; HMT, histone methyltransferase; HKMT, histone lysine methyltransferase; IDH, isocitrate dehydrogenases; KDM, lysine demethylase; α-KG, α-ketoglutarate; MAO, monoamine oxidase; MLL, mixed lineage leukemia; LSD1, lysine-specific demethylase 1; PHF, plant homeodomain finger; PRC2, polycomb repressive complex 2; PRMT, protein arginine methyltransferase; SAH, S-adenosylhomocysteine; SAM, S-adenosylmethionine; SET, su(var)3-9, Enhancer-of-zeste, trithorax; WT, wild-type
